# Characterisation of the androgen regulation of glycine *N*-methyltransferase in prostate cancer cells

**DOI:** 10.1530/JME-13-0169

**Published:** 2013-12

**Authors:** Silvia Ottaviani, Greg N Brooke, Ciara O'Hanlon-Brown, Jonathan Waxman, Simak Ali, Laki Buluwela

**Affiliations:** 1 Department of Surgery and CancerImperial College London, Imperial Centre for Translational and Experimental MedicineLondon, W12 0NNUK; 2 School of Biological SciencesUniversity of EssexColchesterUK

**Keywords:** prostate cancer, androgen receptor, glycine *N*-methyltransferase, androgen response element

## Abstract

The development and growth of prostate cancer is dependent on androgens; thus, the identification of androgen-regulated genes in prostate cancer cells is vital for defining the mechanisms of prostate cancer development and progression and developing new markers and targets for prostate cancer treatment. Glycine *N*-methyltransferase (GNMT) is a *S*-adenosylmethionine-dependent methyltransferase that has been recently identified as a novel androgen-regulated gene in prostate cancer cells. Although the importance of this protein in prostate cancer progression has been extensively addressed, little is known about the mechanism of its androgen regulation. Here, we show that GNMT expression is stimulated by androgen in androgen receptor (AR) expressing cells and that the stimulation occurs at the mRNA and protein levels. We have identified an androgen response element within the first exon of the *GNMT* gene and demonstrated that AR binds to this element *in vitro* and *in vivo*. Together, these studies identify GNMT as a direct transcriptional target of the AR. As this is an evolutionarily conserved regulatory element, this highlights androgen regulation as an important feature of GNMT regulation.

## Introduction

Androgens play a central role in the biology of normal prostate development and prostate cancer progression (Shen & Abate-Shen 2010). These hormones mediate their effects through the action of the androgen receptor (AR), a member of the nuclear receptor superfamily of ligand-activated transcription factors (Brinkmann *et al*. 1999, Lu *et al*. 2006, Dehm & Tindall 2007). Following androgen binding, AR dissociates from heat-shock proteins and translocates to the nucleus where it binds to androgen response elements (AREs) of target genes in association with coactivators and corepressors to regulate gene expression (Shang *et al*. 2002, Heemers & Tindall 2007). Previous studies have extensively shown that transcriptional regulation by AR drives prostatic differentiation during development (Cunha *et al*. 2004) and oncogenic transformation during cancer (Heinlein & Chang 2004, Lamont & Tindall 2011). Therefore, the mainstay treatment for non-organ confined prostate cancer has been directed at suppressing AR activity through androgen deprivation therapies. However, although initially effective, these treatments inevitably fail and, in most cases, the tumours progress to a castration-resistant form, for which few therapeutic options are available. Importantly, accumulated evidence suggests that castration-resistant prostate cancer (CRPC) remain dependent on the expression and transcriptional activity of the AR (Feldman & Feldman 2001, Gregory *et al*. 2001, Chen *et al*. 2004, Balk & Knudsen 2008, Waltering *et al*. 2009). Downstream targets of the AR are therefore of great importance for further characterising the disease and thus developing new markers and therapy targets for prostate cancer treatment.

Glycine *N*-methyltransferase (GNMT) is a tetrameric multifunctional protein that plays an important role in the methionine and one-carbon metabolism by acting as both a methyltransferase enzyme and a folate-binding protein (Blumenstein & Williams 1960, Cook & Wagner 1984). As a methyltransferase, GNMT catalyses the transfer of a methyl group from *S*-adenosylmethionine to glycine to form *S*-adenosylhomocysteine and sarcosine (*N*-methylglycine). In recent years, increasing attention has been paid to the role of GNMT in prostate cancer since the discovery that sarcosine, the metabolite generated by GNMT, was highly elevated during prostate cancer progression to metastasis and, importantly, that it could be detected noninvasively in urine (Sreekumar *et al*. 2009). By its function in generating sarcosine, GNMT was predicted to play a critical role in modulating prostate cancer invasion. Indeed, Sreekumar *et al*. showed that GNMT knockdown resulted in a significant reduction in prostate cancer cell invasion. In addition, experiments carried out in prostate cancer cell lines revealed that androgen treatment up-regulates *GNMT* expression and that the AR directly binds to the *GNMT* promoter (Sreekumar *et al*. 2009).

We have previously characterised the androgen-regulated gene expression in LNCaP cells by microarray analysis and found that *GNMT* was significantly up-regulated following androgen treatment (Ngan *et al*. 2009). Later studies have further explored the role of this protein in prostate cancer (Song *et al*. 2011, Khan *et al*. 2013); however, the androgen regulation of GNMT remained largely unexplored. In light of the important role of this protein in prostate cancer progression, we aimed to characterise further the mechanism of GNMT androgen regulation.

## Materials and methods

### Reagents

The synthetic androgen methyltrienolone (R1881) was purchased from Perkin Elmer Life Sciences (Beaconsfield, UK) and was dissolved in 100% ethanol at a stock concentration of 100 mM and stored at −20 °C. A working concentration of 10 μM was prepared and was added to cells at a final concentration of 1 nM, unless otherwise stated.

### Cell culture

The cell lines used were obtained from the American Type Culture Collection except BPH-1 obtained from Dr Charlotte Bevan (Imperial College, London, UK) and LNCaP-C4 and LNCaP-C4-2B cells obtained from Dr Hayley Whitaker (Cambridge Research Institute, Cambridge, UK). All cell lines were verified by short-tandem repeat profiling (LGC Standards, Teddington, UK) used within 30 passages of the original, source passage and tested every 3 months to ensure mycoplasma negativity (MycoAlert, Lonza, UK).

LNCaP, LNCaP-C4, LNCaP-C4-2B, BPH-1, Du145 and PC-3 cells were maintained at 37 °C, 5% CO_2_ in RPMI-1640 medium (Sigma–Aldrich) with 10% FCS (First Link, Birmingham, UK). T47D and COS-1 cells were maintained in DMEM medium (Sigma–Aldrich) with 10% FCS. RWPE-1 cells were maintained in keratinocyte serum-free medium supplemented with bovine pituitary extract and recombinant epidermal growth factor (Gibco-Invitrogen Corporation). Seventy-two hours before exposure to androgen, media were replaced with phenol red-free RPMI (or DMEM) (Gibco-Invitrogen Corporation) supplemented with 10% dextran-coated charcoal-treated serum (DSS; First Link). All media were supplemented with 2 mM l-glutamine, 100 U/ml penicillin and 100 mg/ml streptomycin (Sigma–Aldrich).

### Plasmids

The *Renilla* luciferase vector was pRL-TK (Promega). AR expression and control vectors (pSG5-AR and pSG5) were gifts from Dr Charlotte Bevan.

To generate the *GNMT* promoter vector, a 1.45 kb *GNMT* promoter region was amplified from LNCaP genomic DNA using the LongRange PCR kit (Qiagen Ltd) (forward 5′-ATCTCAGGGGATGGA-3′, reverse 5′-AAGCAGCCATGCCTT-3′). The PCR product was amplified once again using the LongRange PCR kit with the addition of BglII and HindIII restriction sites (forward 5′-GCTAGGAGATCTCGCGAGAGCGGCCCTGTAATTGAGCAGAAAGG-3′, reverse 5′-CTAGCCAAGCTTCCGCCACCCCCAGGGAGCGGGTCCGGTAC-3′). Both the PCR product and the pGL3-basic vector (Promega) were digested with HindIII and BglII restriction enzymes before ligation and subsequently verified by sequencing.

The *GNMT* promoter ARE mutants were generated by site-directed mutagenesis of the *GNMT* promoter reporter wild-type vector using the QuikChange Multi-site-directed Mutagenesis kit (Agilent Technologies, Stockport, UK). Mutagenic oligonucleotides were designed such that the ARE consensus sequences were abolished by the insertion of a restriction site for the enzyme MluI. The sequences are given in Supplementary Table 1, see section on supplementary data given at the end of this article.

### siRNA transfections

Cells were transfected with control siRNA (Negative control N.2, Ambion, Applied Biosystems) or siRNA specific to AR (s1539, Ambion, Applied Biosystems) using Lipofectamine RNAiMAX (Invitrogen) according to the manufacturer's protocols. LNCaP cells were seeded in 10 cm dishes at a density of 2×10^6^ in 10 ml phenol red-free RPMI supplemented with 10% DSS without antibiotics for 24 h. On the day of transfection, the siRNA transfection reagent complex was prepared by diluting 600 pmol siRNA. This was followed by the addition of Lipofectamine RNAiMAX, mixed gently and incubated for 20 min at room temperature. The siRNA-Lipofectamine complexes were added drop-wise to the cells. Cells were gently mixed and incubated for 24 h, following which fresh phenol red-free RPMI supplemented with 10% DSS and 1 nM R1881 was added. Cells were incubated for 48 h before harvesting for RNA or protein extraction.

### Real-time quantitative PCR

Cells were treated for the indicated times and RNA harvested using RNeasy mini preparation kit (Qiagen Ltd) according to the manufacturer's instructions. Prior to elution, columns were treated with DNase using the RNase-Free DNase Set (Qiagen Ltd) to remove any residual DNA. Two micrograms of RNA were used for RT reaction using RevertAid M-MuLV Reverse Transcriptase (Fermentas, York, UK). The obtained cDNA was then diluted 1:10 and 2 μl cDNA subsequently used as a template for each PCR. TaqMan real-time RT-PCR was carried out according to the manufacturer's instructions on an Applied Biosystems 7500 fast Real-time PCR system using Assay-on Demand primers (Applied Biosystems). The assay identification numbers are given in Supplementary Table 2, see section on supplementary data given at the end of this article.

### Western blotting

Whole cell lysates were prepared in RIPA buffer (Sigma–Aldrich) containing complete protease inhibitors (PIs; Roche Diagnostics Ltd) and protein concentration determined using the Pierce BCA Protein Assay Kit (Thermo Fisher Scientific UK Ltd, Leicestershire, UK). Twenty micrograms of proteins were separated on a 12% SDS–polyacrylamide gel and transferred onto a nitrocellulose membrane for immunodetection, using the iBlot 7-Minute Blotting System (Invitrogen). The membrane was then blocked in PBS-0.1% Tween (v/v) (PBST) containing 5% (w/v) dried skimmed milk powder followed by overnight incubation at 4 °C with gentle shaking with primary antibody against: GNMT (HPA027501, Sigma–Aldrich), AR (Sc-816, Santa Cruz Biotechnologies) and β-actin (ab6276, Abcam Ltd, Cambridge, UK). The membrane was washed three times in PBST and incubated with the appropriate HRP-conjugated secondary antibody (Dako, Ely, UK) for 90 min at room temperature. The membrane was washed again three times in PBST. The SuperSignal West Pico Chemiluminescent Substrate (Perbio Science, Cramlington, UK) was added to the membrane followed by autoradiography using Hyperfilm ECL (GE Healthcare, Chalfont St Giles, UK).

### Confocal microscopy

LNCaP cells grown on glass coverslips were fixed in 4% paraformaldehyde for 10 min at room temperature. Cells were then washed with PBS twice and 0.5 M glycine solution was added for 20 min to quench aldehyde-induced autofluorescence. After washing, cells were permeabilised in 0.3% (v/v) Triton X-100 for 10 min. Coverslips were blocked with 1% (w/v) BSA and 2% FCS (v/v) for 30 min following incubation with the appropriate primary antibody for 1 h at room temperature. Cells were then washed with PBS twice and incubated in the dark for 1 h at room temperature with the secondary antibody Alexa Fluor 488 (Invitrogen). Finally, counterstaining of nuclei was performed using TO-PRO-3 (Invitrogen) for 5 min. The cells were washed again three times and mounted in Vectashield Mounting Medium H-1000 (Vector Laboratories, Southgate, UK). Images were acquired with a Zeiss LSM 510 META confocal microscope fitted with an LSM 510 META scanhead and driven by Zeiss LSM 510 confocal software using a Plan-Apochromat 63× (1.40 numerical aperture, oil) lens and Immersol 518F oil (Carl Zeiss Ltd, Hertfordshire, UK).

### Reporter gene assays

LNCaP cells were seeded in 24-well plates at a density of 70 000 cells/well phenol red-free RPMI medium supplemented with 10% DSS. Forty-eight hours later, the cell medium was replaced with fresh RPMI phenol red-free medium supplemented with 10% DSS with or without 1 nM R1881. Each well was transfected with 500 ng DNA, consisting of: 100 ng of firefly luciferase pGL3 vectors, 100 ng *Renilla* luciferase vector (pRL-TK) and 300 ng Bluescribe plasmid DNA as carrier DNA. Cells were transfected using Lipofectamine LTX and PLUS Reagent (Invitrogen) according to the manufacturer's instructions. Twenty-four hours following transfection, firefly and *Renilla* luciferase activities were determined using the Dual-Glo Luciferase Assay System (Promega). Firefly luciferase readings were subsequently normalized against the control *Renilla* luciferase such that the *Renilla* luciferase activity served to control for transfection efficiency.

### Electrophoretic mobility shift assay

COS-1 cells were seeded in 10 cm dishes at a density of 1×10^6^ in 10 ml DMEM containing 10% FCS without antibiotics. Following overnight incubation, cell medium was replaced with fresh DMEM containing 10% FCS without antibiotics. Cells were transfected using Lipofectamine LTX and PLUS Reagent with 2 μg pSG5 or pSG5-AR expression vectors. Twenty-four hours after transfection, cells were treated with 10 nM R1881 for 1 h. Cells were then washed twice with ice-cold PBS and harvested by scraping in ice-cold PBS buffer containing PIs. Cells were centrifuged at 10 000 ***g*** for 10 min at 4 °C and the pellet re-suspended in high-salt buffer (HSB: 400 mM KCl; 20 mM Tris–HCl, pH 7.5; 2 mM dithiothreitol and 20% glycerol (v/v)) supplemented with PIs. Cells were frozen at −80 °C and thawed on ice three times and then centrifuged for 15 min at 4 °C. The supernatant was stored in aliquots at −80 °C. The double-stranded oligonucleotides containing the ARE sequences are given in Supplementary Table 3, see section on supplementary data given at the end of this article. The oligos were purchased as single strands, one labelled with the infrared IR-800 dye and the other unlabelled (Eurofins, Ebersberg, Germany). Hybridisation of the oligos was performed by mixing 1 μM labelled oligo with 1 μM unlabelled oligo. The mix was placed in boiling water, which was allowed to cool at room temperature overnight. For electrophoretic mobility shift assay (EMSA), 2.5 μl COS-1 HSB extract were pre-incubated with 142 ng/μl poly(deoxyinosine-deoxycytosine) (dI·dC) for 30 min at 4 °C. For supershift, 2 μg AR antibody (sc-816X, ChIP-grade; Santa Cruz Biotechnologies) was added. The hybridised oligos (0.1 μM) were then added followed by incubation for 4 h at 4 °C. The bound probe was separated from unbound probe by gel electrophoresis in a 4% polyacrylamide gel at 100 V for 1 h at 4 °C. The infrared signal was visualised using the Odyssey (LI-COR) IR imaging system.

### Chromatin immunoprecipitation

LNCaP cells were cultured in RPMI medium lacking phenol red supplemented with 10% DSS for 72 h followed by addition of 10 nM R1881 or ethanol for 1 h. Cells were then cross-linked with 1% formaldehyde (Sigma–Aldrich) for 10 min at room temperature and washed three times with PBS containing PIs. Cells were collected by scraping in PBS with PIs and lysed in lysis buffer (1% SDS, 10 mM EDTA and 50 mM Tris–HCl, pH 8.0) containing PIs for 10 min on ice (200 μl lysis buffer/1×10^6^ cells). Cells were sonicated for 10 min at 4 °C using 20-s high-power pulses and then centrifuged at 10 000 ***g*** for 10 min at 4 °C. 1.8 ml dilution buffer (1% Triton X-100, 2 mM EDTA, 20 mM Tris–HCl, pH 8.0, and 150 mM NaCl) was added for 200 μl sonicated lysate. Fifty microliters of sonicated lysate were used as the input sample and the rest was used for immunoprecipitation. Dynabeads protein A (for rabbit IgG1) (Invitrogen) were washed three times with blocking solution (0.5% (w/v) BSA in PBS) and then added to the sonicated lysates for 30 min at 4 °C on rotation. Two micrograms of AR antibody (sc-816X, ChIP-grade; Santa Cruz Biotechnologies) or rabbit IgG were added and incubated overnight at 4 °C on rotation. Preblocked dynabeads were then added and incubated for 1 h at 4 °C on rotation. The supernatant was removed and sequentially the following buffers were added for 5 min on rotation at 4 °C: i) low-salt wash (0.1% SDS, 1% Triton X-100, 2 mM EDTA, 20 mM Tris–HCl, pH 8.0, and 150 mM NaCl)+PIs; ii) high-salt wash (0.1% SDS, 1% Triton X-100, 2 mM EDTA, 20 mM Tris–HCl, pH 8.0, and 500 mM NaCl)+PIs; iii) LiCl wash (0.25 M LiCl, 1% NP40, 1% sodium deoxycholate, 1 mM EDTA and 10 mM Tris–HCl, pH 8.0)+ PIs; and iv) Tris–EDTA wash (10 mM Tris–HCl and 1 mM Na EDTA, pH 8.0). Finally, elution buffer (1% (w/v) SDS, 10 mM NaHCO_3_) was added for 15 min on a shaker at room temperature and 5 M NaCl was added. Reverse cross-linking was performed at 65 °C overnight. 0.5 M EDTA, 1 M Tris–HCl (pH 6.5) and proteinase K were added to each sample and the tubes were then incubated at 45 °C for 1 h. Immunoprecipitated DNA was recovered by phenol/chloroform extraction and quantitative real-time PCR was used to quantify recruitment of AR on *PSA* and *GNMT* promoters. Primers were designed at either side of the *PSA* enhancer or negative control region and the *GNMT* ARE. Primer sequences are given in Supplementary Table 4, see section on supplementary data given at the end of this article.

## Results

### GNMT is up-regulated by androgen treatment in AR-positive cancer cells

**Figure 1:**
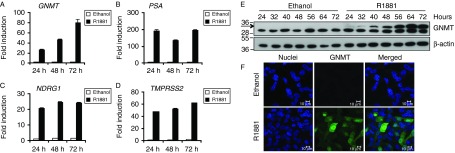
Androgen up-regulates the expression of GNMT mRNA and protein in a time-dependent manner. TaqMan RT-PCR for *GNMT* (A), *PSA* (B), *NDRG1* (C) and *TMPRSS2* (D) was performed from RNA prepared from LNCaP cells cultured in RPMI lacking phenol red supplemented with 10% DSS for 72 h followed by the addition of either ethanol or 1 nM R1881 for 24, 48 and 72 h. Data were normalised to *GAPDH* levels. The expression of each gene in cells treated with ethanol for 24 h was set to one, with the expression level in the other conditions being shown relative to this. Results are shown as mean values of three replicates with error bars showing s.e.m. (E) LNCaP cells were treated for the indicated times, whole cell lysates were separated by SDS–PAGE and immunoblotted for GNMT and β-actin. The polypeptide corresponding to GNMT is arrowed and molecular weight markers are expressed in kDa. (F) Immunofluorescence staining of GNMT was performed in LNCaP cells treated for 48 h. The GNMT antibody was detected with Alexa Fluor 488-labelled secondary and TO-PRO-3 was used for counterstaining of nuclei. All images were acquired using a Zeiss LSM510 confocal microscope. Scale bar=10 μm.

We first examined the mRNA expression of *GNMT* in the LNCaP, AR-positive prostate cancer cell line, which is dependent on androgens for growth. LNCaP cells were maintained in steroid-depleted medium for 72 h and then treated with either ethanol or the synthetic androgen R1881 for 24, 48 and 72 h. *GNMT* expression was then quantified by TaqMan real-time RT-PCR (Fig. 1A). The *GNMT* transcript was stimulated by R1881 in a time-dependent manner, such that a 26-fold increase was detected 24 h after treatment and reached ∼80-fold at 72 h. As a control, expression of the well-known androgen-regulated genes *PSA*, *NDRG1* and *TMPRSS2* was evaluated and up-regulation of their transcripts following androgen treatment was confirmed (Fig. 1B, C, and D). In addition, androgen-dependent up-regulation of GNMT in LNCaP cells was also shown at a protein level, as demonstrated by western blotting (Fig. 1E) and immunofluorescent staining (Fig. 1F).

**Figure 2:**
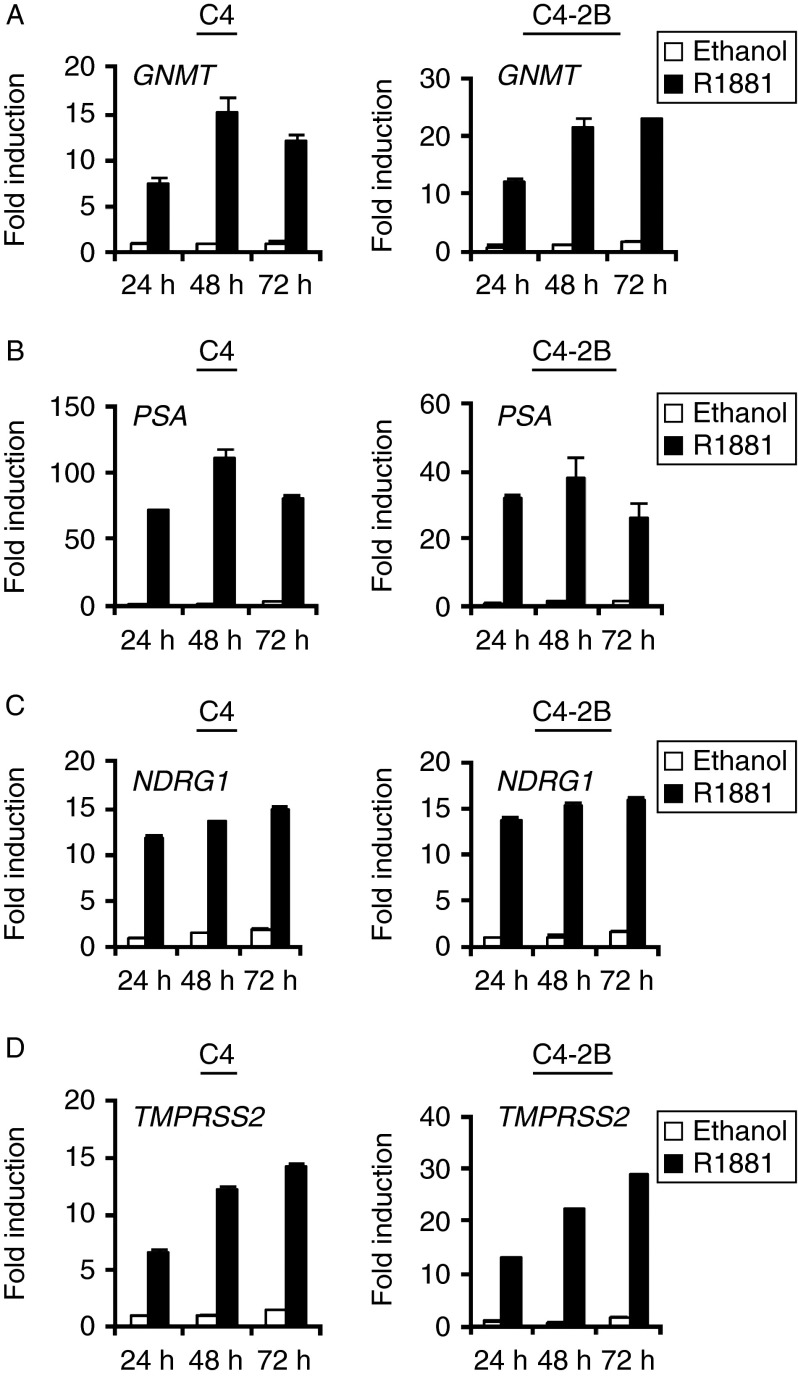
*GNMT* is androgen up-regulated in AR-positive cell lines. TaqMan RT-PCR for *GNMT* (A), *PSA* (B), *NDRG1* (C) and *TMPRSS2* (D) was performed using RNA prepared from LNCaP-derived lines C4 and C4-2B. Cells were cultured and treated using the same conditions described for LNCaP cells in Fig. 1.

To exclude the possibility that GNMT androgen regulation was unique to LNCaP cells, its expression was evaluated in two additional AR-positive cell lines, the hormone-insensitive LNCaP-derived prostate cancer cell lines C4 and C4-2B. Indeed, all cell lines showed androgen regulation of *GNMT* (Fig. 2A) and demonstrated increasing expression of the known androgen-regulated genes *PSA*, *NDRG1* and *TMPRSS2* upon R1881 treatment (Fig. 2B, C, and D). In addition, *GNMT* expression was evaluated in a broader panel of prostate cell lines (Supplementary Figure 1, see section on supplementary data given at the end of this article). The benign prostate epithelial RWPE-1 cell line, the benign prostatic hyperplasia-derived cell line BPH-1, as well as DU145 and PC-3 cell lines showed low or undetectable levels for *AR* and low levels of *GNMT*. It was therefore clear that the expression of *GNMT* was limited to prostate lines expressing high levels of *AR*.

### AR knockdown inhibits GNMT expression in LNCaP cells

**Figure 3:**
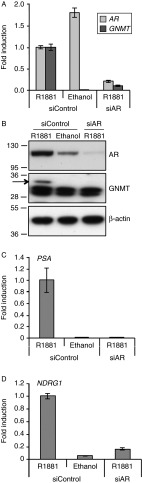
*AR* knockdown suppresses the androgen-induced *GNMT* gene. (A) LNCaP cells were cultured in RPMI containing 10% DSS in the absence of antibiotics for 24 h. Cells were then transfected with siRNAs targeting AR or control. Twenty-four hours after transfection, cells were treated with 1 nM R1881 with an equal volume of ethanol being added to the vehicle control. RNA was prepared after a further 48 h and TaqMan RT-PCR for *AR* and *GNMT* was performed. Data have been normalised to *GAPDH* levels and *AR* or *GNMT* expression levels in the siRNA control treated with R1881 was set to one. Results are shown as mean values of three independent experiments performed in triplicates with error bars representing s.e.m. (B) LNCaP cells were transfected with siRNAs as for A. Whole cell lysates were prepared and immunoblotted for AR, GNMT (arrowed) and β-actin. Molecular weight markers are expressed in kDa. Suppression of the androgen-regulated genes *PSA* (C) and *NDRG1* (D) following silencing of AR is also shown.

To determine whether the androgen regulation of GNMT was mediated by AR, we performed RNA interference of AR in LNCaP cells. LNCaP cells were maintained in steroid-depleted medium for 24 h and subsequently transfected with control and AR-specific siRNAs. Twenty-four hours after transfection, 1 nM R1881 or ethanol was added for a further 48 h and RNA and proteins were collected. Interestingly, we found that AR knockdown significantly suppressed *GNMT* mRNA (Fig. 3A) and protein levels (Fig. 3B) in the presence of androgen, suggesting that GNMT is a transcriptional target of AR. To confirm the specificity of the siRNA targeting AR, we also showed the repression of *AR* mRNA and protein upon knockdown. Notably, we observed a significant reduction of *AR* mRNA level upon R1881 treatment, which is in agreement with the finding that *AR* mRNA expression shows a biphasic regulation by androgen (Nirde *et al*. 1998) through two AREs in exon 5 of the *AR* gene (Dai & Burnstein 1996).

As a control, we demonstrated reduction of the well-known androgen-regulated genes *PSA* (Fig. 3C) and *NDRG1* (Fig. 3D) following silencing of AR. Taken together, these results show that GNMT androgen up-regulation is mediated by AR.

### Identification of an ARE in exon 1 of the *GNMT* gene

As GNMT expression is highly responsive to R1881, we hypothesised that AR was directly regulating its expression. By carrying out a bioinformatic analysis for steroid hormone response elements using the web-based tool Mulan (http://mulan.dcode.org/) (Ovcharenko *et al*. 2005), three putative AREs were identified proximal to the gene promoter region: ARE-I and ARE-II at position −1111/−1097 and −716/−708 respectively, and ARE-III at position +19/+33 (Supplementary Figure 2, see section on supplementary data given at the end of this article). The position of the start site at the 14th base upstream the ATG codon was identified by Lee *et al*. (2009) and confirmed by 5′ RACE analysis of androgen-treated LNCaP mRNA (data not shown).

**Figure 4:**
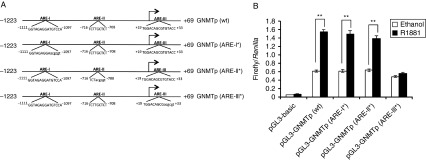
Site-directed mutagenesis analysis of the three predicted AREs. (A) The wild-type *GNMT* promoter reporter construct was generated by cloning a 1.2 kb region of the *GNMT* gene into a pGL3-basic luciferase vector. This construct was used as a template for site-directed mutagenesis of ARE-I (ARE-I*), ARE-II (ARE-II*) and ARE-III (ARE-III*). The mutants were generated by replacing part of the ARE sequence with the MluI restriction site sequence (5′-acggct-3′). (B) The activity of the *GNMT* ARE mutants was tested using the luciferase reporter assay. Firefly luciferase activities were normalised for transfection efficiency against the *Renilla* luciferase activities. Results are shown as mean of three independent experiments, each performed in triplicate. Error bars represent the s.e.m. Statistical significance was calculated by unpaired two-tailed Student's *t*-test: **P<0.01.

To investigate the function of these AREs, we carried out a gene promoter analysis. In brief, a region of the *GNMT* gene that includes the three putative AREs was amplified by PCR from LNCaP genomic DNA and cloned into the pGL3-basic reporter vector, upstream of the promoter-less firefly luciferase gene (GNMTp). In addition, we generated reporter constructs containing mutations in the three AREs by site-directed mutagenesis (Fig. 4A). The androgen responsiveness of the wild-type and mutated *GNMT* promoter constructs was determined by luciferase reporter assays. LNCaP cells were transiently transfected with the luciferase constructs in the absence or presence of androgen. The wild-type *GNMT* reporter constructs showed stimulation of the luciferase gene upon R1881 treatment of approximately 2-fold. Mutation of ARE-I and -II did not show a decrease in androgen responsiveness, whereas mutation of ARE-III resulted in an ablation of androgen-inducible luciferase activity, indicating that AR directly regulates *GNMT* expression through this ARE (Fig. 4B).

### AR binds to the *GNMT* ARE in an androgen-dependent manner *in vitro* and *in vivo*

**Figure 5:**
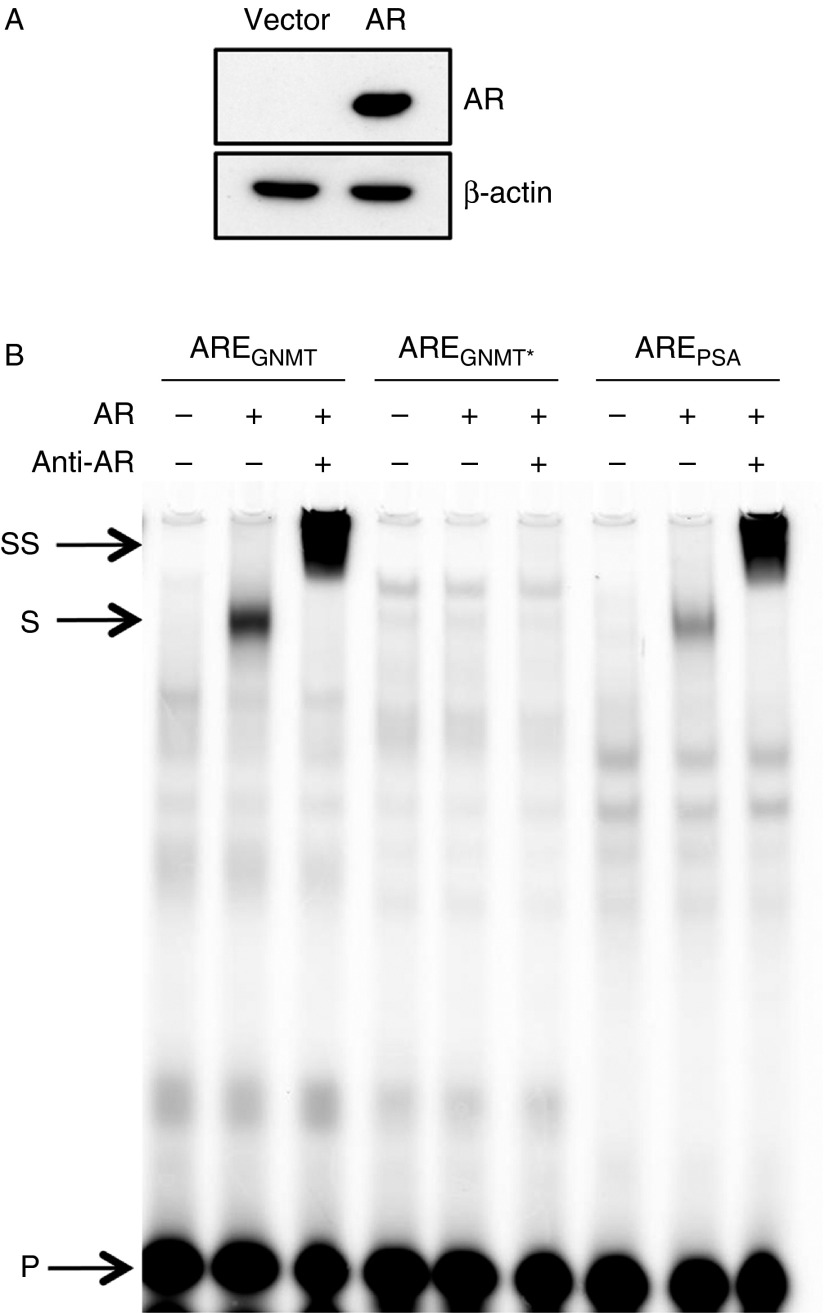
EMSA shows that AR binds to the ARE located within exon 1 of the *GNMT* gene. (A) COS-1 cells cultured in DMEM containing 10% FCS in the absence of antibiotics were transiently transfected with pSG5 control vector or AR expression plasmids. Twenty-four hours after transfection, cells were treated for 1 h with 10 nM R1881. High-salt buffer (HSB) cell extracts were prepared as described in the Materials and methods section. AR expression was confirmed by western blotting. (B) HSB cell extracts were first incubated with poly(dI·dC), in the absence or presence of an AR antibody, followed by incubation with the IR-800-labelled double-stranded oligonucleotides. The extract–oligonucleotide mix was resolved by electrophoresis through a 4% polyacrylamide gel and migration of the oligonucleotides through the gel was determined using the Odyssey (LI-COR) IR imaging system. Arrows indicate the positions of the unbound (P), shifted (S) and the antibody-AR-oligonucleotide ‘supershifted’ (SS) probe.

We then assessed whether AR was able to bind to this sequence *in vitro* by performing EMSA. The sequence of the wild-type and mutant *GNMT* AREs together with the ARE-I contained in the *PSA* promoter was synthesized as oligonucleotides having the 5′ end labelled with the infrared IR-800 dye (Supplementary Table 3). The *PSA* oligonucleotide sequence was chosen as positive control, as it has been previously shown to specifically bind the AR in EMSA assays (Luke & Coffey 1994). EMSA was performed using cell extracts from COS-1 cells transfected with either AR expression vector or pSG5 empty vector (Fig. 5A) and revealed that the AR interacted with the *GNMT* as well as the *PSA* AREs, both in the absence and presence of an AR antibody, whereas no binding was detected to the mutated *GNMT* ARE (Fig. 5B).

**Figure 6:**
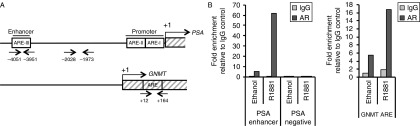
AR is recruited to the *GNMT* ARE in an androgen-dependent manner. (A) Diagramatic representation of the *PSA* (top) and *GNMT* (bottom) promoter regions. AREs are highlighted in boxes and arrows indicate the location of PCR primer pairs. (B) LNCaP cells were cultured in RPMI medium lacking phenol red supplemented with 10% DSS for 72 h followed by addition of 10 nM R1881 or ethanol for 1 h. Cells were then cross-linked, lysed and sonicated. Immunoprecipitations were performed using an antibody specific for AR or rabbit IgG. Immunoprecipitated DNA was reverse cross-linked and recovered by phenol/chloroform extraction. Real-time PCR was carried out using primers for the regions indicated in part A. The *PSA* enhancer region was chosen as a positive control, and a region distant from ARE elements as a negative control. Results show the mean values obtained from ChIP analysis of two replicates.

Finally, to confirm that AR was recruited on the ARE located within exon 1 of the *GNMT* gene, chromatin immunoprecipitation (ChIP) assay was carried out. LNCaP cells were maintained in steroid-depleted medium for 72 h and subsequently treated with either ethanol or R1881. ChIP was performed using AR antibody or rabbit IgG as a control. AR binding was quantified by real-time PCR using primer pairs surrounding the *GNMT* ARE as well as the enhancer ARE element and a region to which AR does not bind, within the *PSA* gene that provided a positive and negative control respectively (Fig. 6A). The ChIP assay revealed a ligand-dependent recruitment of AR to the *GNMT* ARE (Fig. 6B).

## Discussion

In this study, we have characterised the expression and the androgen regulation of GNMT and identified an ARE in exon 1 of the *GNMT* gene. Sreekumar *et al*. (2009) demonstrated that *GNMT* mRNA increased upon androgen treatment in LNCaP and VCaP cell lines. In agreement with these findings, we have shown that GNMT mRNA and protein are strongly stimulated by the synthetic androgen R1881 in the LNCaP cell line in a time-dependent manner. Indeed, we have previously shown that *GNMT* belongs to a cluster of genes (Cluster U4) whose expression increases slowly over time upon R1881 treatment (Ngan *et al*. 2009). The androgen regulation of GNMT was also confirmed in two additional AR-positive cell lines, the hormone-insensitive LNCaP sublines C4 and C4-2B. Furthermore, we have shown that GNMT stimulation of expression is indeed AR mediated, as knockdown of AR resulted in a reduction in GNMT mRNA and protein levels.

The strong stimulation of GNMT by androgen together with the detection of its expression in AR-positive cancer cell lines and its inhibition upon AR knockdown indicated that AR may directly regulate GNMT expression. As AR regulates transcription by binding to consensus DNA sequences in the regulatory regions of its target genes (Heinlein & Chang 2004), we searched for putative AREs in the *GNMT* promoter region. This analysis resulted in the identification of three predicted AREs: ARE-I and ARE-II, located within the *GNMT* promoter, and ARE-III, located within exon 1 of the *GNMT* gene. A 1.2 kb region of the *GNMT* promoter provided androgen regulation to a luciferase reporter gene. Mutation of ARE-I or ARE-II had no effect on androgen regulation, whereas mutation of ARE-III prevented androgen stimulation, demonstrating that ARE-III is able to function as an ARE. In addition, EMSA analysis showed that the AR can bind to this sequence and ChIP analysis confirmed that AR is recruited in an androgen-dependent manner to a region of the *GNMT* gene encompassing ARE-III.

**Figure 7:**
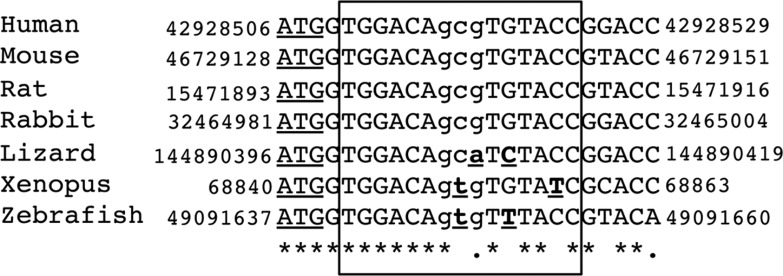
Nucleotide sequences of the region flanking the *GNMT* ARE in multiple species are shown. The box demarks the region of ARE homology and the translation start site is underlined. Sequences were retrieved from the UCSC (http://genome.ucsc.edu) using the following genome builds: hg19 (human), mm10 (mouse), rn5 (rat), oryCun2 (rabbit), anoCar2 (lizard), xenTro3 (xenopus) and danRer7 (zebrafish). Multiple-sequence alignment was performed using Clustal Omega (http://www.ebi.ac.uk/Tools/msa/clustalo/). Any nucleotide change from the human sequence is in bold and underlined. ‘*’ indicates positions that have a single, fully conserved residue; ’.’ indicates conservation between groups of weakly similar properties (scoring≤0.5 in the Gonnet PAM 250 matrix).

The presence of an ARE within the coding region of a gene has also been described for other androgen-regulated genes, including the rat cystatin-related protein (*crp2*) gene (Devos *et al*. 1997) and the human secretory component (*SC*) gene (Haelens *et al*. 1999, 2001). GNMT is a protein that is highly conserved among mammalian species. About 90% amino acid sequence similarity is observed between human, rabbit, rat and pig GNMT proteins (Ogawa *et al*. 1993, 1998). By performing multiple-sequence alignment analysis, we found that the ARE was fully conserved in mammalian species and highly conserved in the vertebrate lineage, suggesting that this response element may act as a functional ARE across species throughout evolution (Fig. 7).

Recent advances in ChIP-based assays have enabled the generation of comprehensive maps of transcription factors binding across the entire genome. With the aim of understanding the AR-regulated gene network in either androgen dependent or CRPC, global AR binding events have been mapped, initially using microarrays of gene promoter regions (Massie *et al*. 2007) and later by genome-tiled arrays (Wang *et al*. 2009) and massively parallel sequencing (Lin *et al*. 2009, Sreekumar *et al*. 2009, Yu *et al*. 2010, Massie *et al*. 2011). Interestingly, the ChIP-seq assay performed by Sreekumar *et al*. revealed that AR was recruited to the *GNMT* proximal promoter in LNCaP and VCaP cell lines. Even though the region is not clearly annotated in the paper, it appears to include the *GNMT* ARE identified in this study, consistent with our findings.

Having identified a functional ARE within the *GNMT* gene, we wondered whether the response element was an example of a so-called ‘classical’ ARE, recognised by all class I receptors, or ‘selective’ ARE, recognised mainly by the AR (Denayer *et al*. 2010). Based on the sequence homology to other previously validated AREs, we were able to classify the *GNMT* ARE as a ‘classical’ ARE (Tables 1 and 2). Consequently, not only AR but also the other class I hormone receptors, glucocorticoid receptor (GR), progestogen receptor and mineralocorticoid receptor, should recognise this sequence. It has previously been shown that the glucocorticoid dexamethasone (DEX) is able to activate GNMT in the rat liver and to stimulate its expression in the rat hepatoma cell line H4IIE (Rowling & Schalinske 2003). However, to date, no glucocorticoid response elements (GREs) have been identified in this promoter region (Lee *et al*. 2009). Therefore, we wondered whether the *GNMT* ARE was also a binding site for GR in ChIP-seq assays. Interestingly, the GR ChIP-seq performed in the A549 lung epithelial carcinoma cell line treated with DEX revealed GR binding in a region comprising the transcription start site and first exon of the *GNMT* gene (Reddy *et al*. 2009). On the basis of these observations, we propose that the *GNMT* ARE identified in this study could also act as a GRE. Further studies will certainly be required to verify this hypothesis, the most obvious of which would be to determine whether GR activates the *GNMT* reporter genes we have described herein.

While we were preparing our manuscript, Lee *et al*. (2013) have reported five predicted ARE motifs in the coding region and main body of the human *GNMT* gene and concluded that the only functional ARE was the 5′ most element, which corresponds to the conserved motif we have also identified. In our studies, we have examined three ARE-like motifs in the *GNMT* promoter region and have concluded that the androgen regulation of GNMT can be imparted by binding of AR to a ‘classical’ ARE located within exon 1 of the *GNMT* gene, 18 bases downstream the *GNMT* gene transcription start site, and overlapping the translation start site. Collectively, these results show GNMT as a direct transcriptional target of the AR in the androgen-dependent LNCaP cell line and suggest that regulation through a highly conserved ARE element is a feature of GNMT expression in the vertebrate lineage.

## Supplementary data

This is linked to the online version of the paper at http://dx.doi.org/10.1530/JME-13-0169.

Supplemental Data

## Figures and Tables

**Table 1:** Examples of genes encoding classical AREs

**Gene**	**Classical AREs** (AGAACA nnn TGTTCT)	**Reference**
*MMTV*	AGAACA gtt TGTAAC	Ham *et al*. (1988)
*C3(1)*	AGAACA tca CGTACT	Claessens *et al*. (1989)
*PSA (ARE-I)*	AGAACA gca AGTGCT	Lund *et al*. (1991)
*PSA (ARE-III)*	GGAACA tat TGTATC	Cleutjens *et al*. (1997)
*GNMT*	TGGACA gcg TGTACC	

**Table 2:** Examples of genes encoding AR-selective AREs

**Gene**	**Selective AREs** (AGAACA nnn AGAACA)	**Reference**
*PB*	AGTACT cca AGAACC	Rennie *et al*. (1993)
*SC (enhancer)*	AGAACT ctg CGAACA	Verrijdt *et al*. (1999)
*SC*	GGGACA cag CCTGCT	Haelens *et al*. (2001)
*SLP*	AGAACT ggc TGACCA	Verrijdt *et al*. (2002)

## References

[bib1] Balk SP, Knudsen KE (2008). AR, the cell cycle, and prostate cancer. Nuclear Receptor Signaling.

[bib2] Blumenstein J, Williams GR (1960). The enzymic *N*-methylation of glycine. Biochemical and Biophysical Research Communications.

[bib3] Brinkmann AO, Blok LJ, de Ruiter PE, Doesburg P, Steketee K, Berrevoets CA, Trapman J (1999). Mechanisms of androgen receptor activation and function. Journal of Steroid Biochemistry and Molecular Biology.

[bib4] Chen CD, Welsbie DS, Tran C, Baek SH, Chen R, Vessella R, Rosenfeld MG, Sawyers CL (2004). Molecular determinants of resistance to antiandrogen therapy. Nature Medicine.

[bib46] Claessens F, Celis L, Peeters B, Heyns W, Verhoeven G, Rombauts W (1989). Functional characterization of an androgen response element in the first intron of the C3(1) gene of prostatic binding protein. Biochemical and Biophysical Research Communications.

[bib47] Cleutjens KB, van der Korput HA, van Eekelen CC, van Rooij HC, Faber PW, Trapman J (1997). An androgen response element in a far upstream enhancer region is essential for high, androgen-regulated activity of the prostate-specific antigen promoter. Molecular Endocrinology.

[bib5] Cook RJ, Wagner C (1984). Glycine *N*-methyltransferase is a folate binding protein of rat liver cytosol. PNAS.

[bib6] Cunha GR, Ricke W, Thomson A, Marker PC, Risbridger G, Hayward SW, Wang YZ, Donjacour AA, Kurita T (2004). Hormonal, cellular, and molecular regulation of normal and neoplastic prostatic development. Journal of Steroid Biochemistry and Molecular Biology.

[bib7] Dai JL, Burnstein KL (1996). Two androgen response elements in the androgen receptor coding region are required for cell-specific up-regulation of receptor messenger RNA. Molecular Endocrinology.

[bib8] Dehm SM, Tindall DJ (2007). Androgen receptor structural and functional elements: role and regulation in prostate cancer. Molecular Endocrinology.

[bib9] Denayer S, Helsen C, Thorrez L, Haelens A, Claessens F (2010). The rules of DNA recognition by the androgen receptor. Molecular Endocrinology.

[bib10] Devos A, Claessens F, Alen P, Winderickx J, Heyns W, Rombauts W, Peeters B (1997). Identification of a functional androgen-response element in the exon 1-coding sequence of the cystatin-related protein gene crp2. Molecular Endocrinology.

[bib11] Feldman BJ, Feldman D (2001). The development of androgen-independent prostate cancer. Nature Reviews. Cancer.

[bib12] Gregory CW, He B, Johnson RT, Ford OH, Mohler JL, French FS, Wilson EM (2001). A mechanism for androgen receptor-mediated prostate cancer recurrence after androgen deprivation therapy. Cancer Research.

[bib13] Haelens A, Verrijdt G, Schoenmakers E, Alen P, Peeters B, Rombauts W, Claessens F (1999). The first exon of the human sc gene contains an androgen responsive unit and an interferon regulatory factor element. Molecular and Cellular Endocrinology.

[bib14] Haelens A, Verrijdt G, Callewaert L, Peeters B, Rombauts W, Claessens F (2001). Androgen-receptor-specific DNA binding to an element in the first exon of the human secretory component gene. Biochemical Journal.

[bib48] Ham J, Thomson A, Needham M, Webb P, Parker M (1988). Characterization of response elements for androgens, glucocorticoids and progestins in mouse mammary tumour virus. Nucleic Acids Research.

[bib15] Heemers HV, Tindall DJ (2007). Androgen receptor (AR) coregulators: a diversity of functions converging on and regulating the AR transcriptional complex. Endocrine Reviews.

[bib16] Heinlein CA, Chang C (2004). Androgen receptor in prostate cancer. Endocrine Reviews.

[bib17] Khan AP, Rajendiran TM, Ateeq B, Asangani IA, Athanikar JN, Yocum AK, Mehra R, Siddiqui J, Palapattu G, Wei JT (2013). The role of sarcosine metabolism in prostate cancer progression. Neoplasia.

[bib18] Lamont KR, Tindall DJ (2011). Minireview: alternative activation pathways for the androgen receptor in prostate cancer. Molecular Endocrinology.

[bib19] Lee CM, Shih YP, Wu CH, Chen YM (2009). Characterization of the 5′ regulatory region of the human glycine *N*-methyltransferase gene. Gene.

[bib20] Lee CM, Yen CH, Tzeng TY, Huang YZ, Chou KH, Chang TJ, Chen YM (2013). Androgen response element of the glycine *N*-methyltransferase gene is located in the coding region of its first exon. Bioscience Reports.

[bib21] Lin B, Wang J, Hong X, Yan X, Hwang D, Cho JH, Yi D, Utleg AG, Fang X, Schones DE (2009). Integrated expression profiling and ChIP-seq analyses of the growth inhibition response program of the androgen receptor. PLoS ONE.

[bib22] Lu NZ, Wardell SE, Burnstein KL, Defranco D, Fuller PJ, Giguere V, Hochberg RB, McKay L, Renoir JM, Weigel NL (2006). International Union of Pharmacology. LXV. The pharmacology and classification of the nuclear receptor superfamily: glucocorticoid, mineralocorticoid, progesterone, and androgen receptors. Pharmacological Reviews.

[bib23] Luke MC, Coffey DS (1994). Human androgen receptor binding to the androgen response element of prostate specific antigen. Journal of Andrology.

[bib60] Lund SD, Gallagher PM, Wang B, Porter SC, Ganschow RE (1991). Androgen responsiveness of the murine beta-glucuronidase gene is associated with nuclease hypersensitivity, protein binding, and haplotype-specific sequence diversity within intron 9. Molecular and Cellular Biology.

[bib24] Massie CE, Adryan B, Barbosa-Morais NL, Lynch AG, Tran MG, Neal DE, Mills IG (2007). New androgen receptor genomic targets show an interaction with the ETS1 transcription factor. EMBO Reports.

[bib25] Massie CE, Lynch A, Ramos-Montoya A, Boren J, Stark R, Fazli L, Warren A, Scott H, Madhu B, Sharma N (2011). The androgen receptor fuels prostate cancer by regulating central metabolism and biosynthesis. EMBO Journal.

[bib26] Ngan S, Stronach EA, Photiou A, Waxman J, Ali S, Buluwela L (2009). Microarray coupled to quantitative RT-PCR analysis of androgen-regulated genes in human LNCaP prostate cancer cells. Oncogene.

[bib27] Nirde P, Georget V, Terouanne B, Galifer RB, Belon C, Sultan C (1998). Quantitation of androgen receptor messenger RNA from genital skin fibroblasts by reverse transcription – competitive polymerase chain reaction. Journal of Steroid Biochemistry and Molecular Biology.

[bib28] Ogawa H, Gomi T, Fujioka M (1993). Mammalian glycine *N*-methyltransferases. Comparative kinetic and structural properties of the enzymes from human, rat, rabbit and pig livers. Comparative Biochemistry and Physiology, Part B.

[bib29] Ogawa H, Gomi T, Takusagawa F, Fujioka M (1998). Structure, function and physiological role of glycine *N*-methyltransferase. International Journal of Biochemistry & Cell Biology.

[bib30] Ovcharenko I, Loots GG, Giardine BM, Hou M, Ma J, Hardison RC, Stubbs L, Miller W (2005). Mulan: multiple-sequence local alignment and visualization for studying function and evolution. Genome Research.

[bib31] Reddy TE, Pauli F, Sprouse RO, Neff NF, Newberry KM, Garabedian MJ, Myers RM (2009). Genomic determination of the glucocorticoid response reveals unexpected mechanisms of gene regulation. Genome Research.

[bib40] Rennie PS, Bruchovsky N, Leco KJ, Sheppard PC, McQueen SA, Cheng H, Snoek R, Hamel A, Bock ME, MacDonald BS (1993). Characterization of two cis-acting DNA elements involved in the androgen regulation of the probasin gene. Molecular Endocrinology.

[bib32] Rowling MJ, Schalinske KL (2003). Retinoic acid and glucocorticoid treatment induce hepatic glycine *N*-methyltransferase and lower plasma homocysteine concentrations in rats and rat hepatoma cells. Journal of Nutrition.

[bib33] Shang Y, Myers M, Brown M (2002). Formation of the androgen receptor transcription complex. Molecular Cell.

[bib34] Shen MM, Abate-Shen C (2010). Molecular genetics of prostate cancer: new prospects for old challenges. Genes and Development.

[bib35] Song YH, Shiota M, Kuroiwa K, Naito S, Oda Y (2011). The important role of glycine *N*-methyltransferase in the carcinogenesis and progression of prostate cancer. Modern Pathology.

[bib36] Sreekumar A, Poisson LM, Rajendiran TM, Khan AP, Cao Q, Yu J, Laxman B, Mehra R, Lonigro RJ, Li Y (2009). Metabolomic profiles delineate potential role for sarcosine in prostate cancer progression. Nature.

[bib42] Verrijdt G, Schauwaers K, Haelens A, Rombauts W, Claessens F (2002). Functional interplay between two response elements with distinct binding characteristics dictates androgen specificity of the mouse sex-limited protein enhancer. Journal of Biological Chemistry.

[bib41] Verrijdt G, Schoenmakers E, Alen P, Haelens A, Peeters B, Rombauts W, Claessens F (1999). Androgen specificity of a response unit upstream of the human secretory component gene is mediated by differential receptor binding to an essential androgen response element. Molecular Endocrinology.

[bib37] Waltering KK, Helenius MA, Sahu B, Manni V, Linja MJ, Janne OA, Visakorpi T (2009). Increased expression of androgen receptor sensitizes prostate cancer cells to low levels of androgens. Cancer Research.

[bib38] Wang Q, Li W, Zhang Y, Yuan X, Xu K, Yu J, Chen Z, Beroukhim R, Wang H, Lupien M (2009). Androgen receptor regulates a distinct transcription program in androgen-independent prostate cancer. Cell.

[bib39] Yu J, Mani RS, Cao Q, Brenner CJ, Cao X, Wang X, Wu L, Li J, Hu M, Gong Y (2010). An integrated network of androgen receptor, polycomb, and TMPRSS2-ERG gene fusions in prostate cancer progression. Cancer Cell.

