# Supplementary material for: Characterisation of the androgen regulation of glycine N-methyltransferase in prostate cancer cells
Source: J Mol Endocrinol. 2013 Aug 30;51(3):301–12. doi: 10.1530/JME-13-0169 (PMC3821059; doi:10.1530/JME-13-0169)
Supplement: Supplemental Data [file supp_51_3_301__index.html]

Supplemental Data 

# Characterisation of the androgen regulation of glycine *N*-methyltransferase in prostate cancer cells

## Supplementary Data

**Files in this Data Supplement:**

- Supplementary figure 1 - **Supplementary Fig 1** GNMT is expressed exclusively in AR positive cell lines. The breast cancer T47D cell line together with a panel of prostate cell lines were cultured in medium containing 10% FCS. RNA was prepared and TaqMan RT-PCR for *GNMT* and *AR* was performed. *AR* and *GNMT* expression in cell lines is shown relative to the expression in LNCaP cells. Results are shown as mean values of three replicates with error bars showing s.e.m. (PDF 135 KB)
- Supplementary figure 2 - **Supplementary Fig 2** Human *GNMT* gene sequence from promoter region - 1320 bp to + 240 bp (intron 1) is shown. The sequence was retrieved from the UCSC hg19 human genome assembly (http://genome.ucsc.edu). Boxes indicate the three predicted AREs. Exon 1 is shown in uppercase letters, with sequences 5’ to the transcription start and intron 1 sequences shown in lowercase letters. The 5’ untranslated region (UTR) is indicated in blue and the coding region in red. +1 indicates the transcription start site. (PDF 69 KB)
- Supplementary table 1 - Mutagenesis primers used for generating the GNMTp-ARE mutants (PDF 87 KB)
- Supplementary table 2 - TaqMan Assay-on Demand primers (Applied Biosystems) (PDF 102 KB)
- Supplementary table 3 - Oligonucleotide sequences used for the EMSA assay (PDF 88 KB)
- Supplementary table 4 - ChIP primers for real-time PCR (PDF 87 KB)
