# Supplementary material for: Characterisation of the androgen regulation of glycine N-methyltransferase in prostate cancer cells
Source: J Mol Endocrinol. 2013 Aug 30;51(3):301–12. doi: 10.1530/JME-13-0169 (PMC3821059; doi:10.1530/JME-13-0169)
Supplement: Supplemental Data [file supp_JME-13-0169_Supplementary_figure_1.pdf]

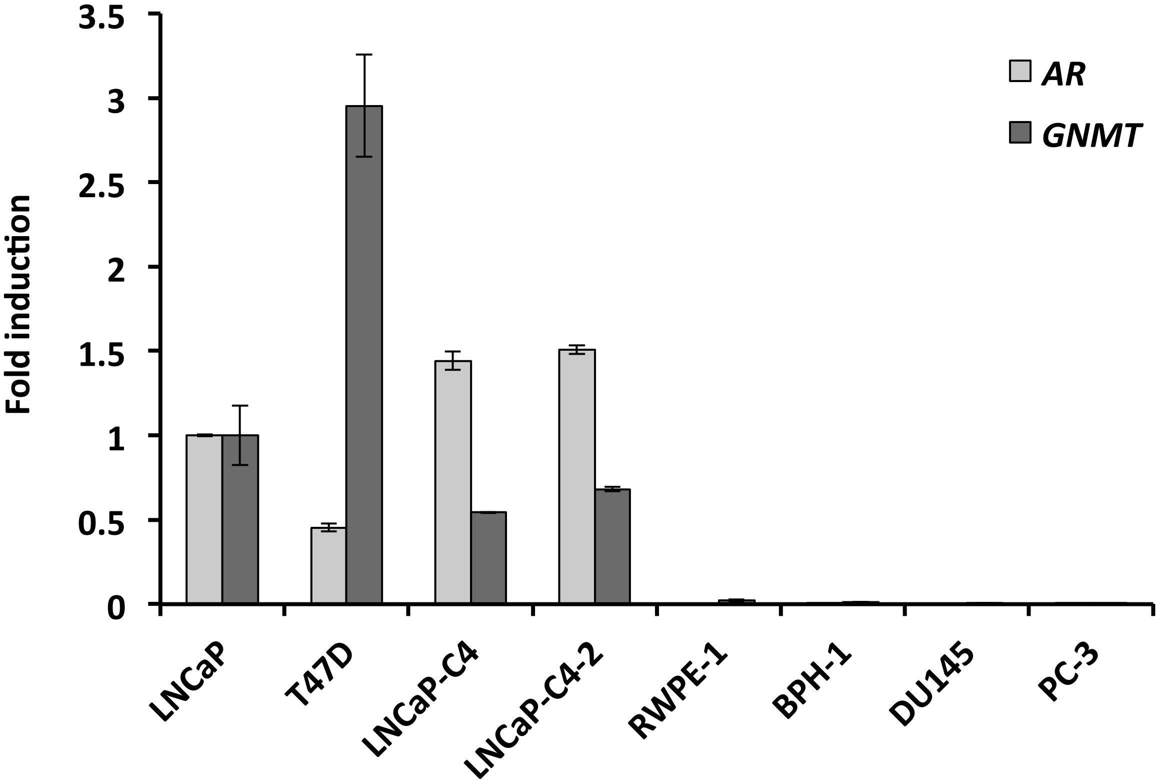

	LNCaP	T47D	LNCaP-C4	LNCaP-C4-2B	RWPE-1	BPH-1	Du145	PC-3
AR Ct mean	20.65	23.18	22.11	21.8	Undetermined	30.11	Undetermined	35.1
GNMT Ct mean	24.05	23.85	26.88	26.3	29.57	29.83	30.86	31.83

Supplementary Fig.1. GNMT is expressed exclusively in AR positive cell lines. The breast cancer T47D cell line together with a panel of prostate cell lines were cultured in medium containing 10% FCS. RNA was prepared and TaqMan RT-PCR for *GNMT* and *AR* was performed. *AR* and *GNMT* expression in cell lines is shown relative to the expression in LNCaP cells. Results are shown as mean values of three replicates with error bars showing s.e.m.
