# Supplementary material for: Characterisation of the androgen regulation of glycine N-methyltransferase in prostate cancer cells
Source: J Mol Endocrinol. 2013 Aug 30;51(3):301–12. doi: 10.1530/JME-13-0169 (PMC3821059; doi:10.1530/JME-13-0169)
Supplement: Supplemental Data [file supp_JME-13-0169_Supplementary_figure_2.pdf]

```
-1320   aaccctgcag ttgcttccat gtttaggcgc tgcccaccaa gggtcccaag ttggaaagga
-1260   aggaggagag aggcagagag agagagagag agagagagag agagagaagc gcgagagcgg
-1200   ccctgtaatt gagcagaaag gaaagggaga aaaatgaatc ccaaactttg ggcttacctc
-1140   tttctgctgg ctggctcgcc aaaatatgtt aacggtagag gatgtccagg ttcttggcat
                                                ARE I
-1080   cttgaacaaa caattggaca aaatgcacaa acaaagcaag aaaggaatga aggattttat
-1020   tgaaaatgaa agtacactcc acagtgtggg agcgagcatg agcatagggg ctcaaaggcc
-960    ctgttacatt ttgtgagttt aaataccctc tacttggggt acaccctatg taaatgaaga
-900    ggatgaagta aagttacagt tacaaagtta ttaatggcgg gcatacgccc tatggagagg
-840    atatttcccg ttataactga agtgtgaatt ggccttatgt tccctgtctc tagaccctat
-780    tctcctgctt cctgtgaaag cagagggttt tgtttgtttt ttgcttttttg tttttgaga
-720    cggagtcttg ctctgttgcc caggctggag tgcagtggtg cactcggctc actgcaactt
              ARE II
-660    ccgccttccc gggttcaagc gattctcctg gctcagcctc cggagtagct aggactacag
-600    gcgcgtgtca ccatgtccca gctaatcttt ctttattttt agtagagacg tgttagccag
-540    gtttcaccgt gttaccgtgt taccgtattc cagccgggct ccgcccctgg attcggtgga
-480    cagaccagct ttacctccag agactccccc tgtcttccct gcaccggctg actataccta
-420    cacacttcgg tggcacaatc tttgtttaga tatgctgatg ccttccttcg ctgggaacgg
-360    tccagtcctg cccgcctggc gctcagctct gcggatctca ggggatggag tggcaatctc
-300    attgctcccg cctctcctga cccctctcgc cttctcctcg agacccgggt taagtgggca
-240    gagggctccg agggtcctct cccgccgtgg gattgcacag agggctgggt ccgcaggctg
-180    gctaaaagga cctagcccag gattgcgagg cagcgccctc cctgcagctg gacagactcg
-120    ggcgcccggc ccaaagcccg gcaggggcgt gtccgcgcgc tcacctgcta ttggccaggt
-60     ggggctgtcg gctgccagca gtgcttatgc tttaagtgcg gagcgggtgg ctgcggagcc
 1      AGGCGCGGCG CAGGATGGTG GACAGCGTGT ACCGGACCCG CTCCCTGGGG GTGGCGGCCG
        +1                   ARE III
 61     AAGGGCTCCC GGACCAGTAC GCGGACGGGG AGGCGGCGCG CGTGTGGCAG CTGTATATCG
 121    GAGACACCCG CAGCCGCACC GCCGAGTACA AGGCATGGCT GCTTGGGCTG CTGCGCCAGC
 181    ACGGCTGCCA GCGGGTGCTC GACGTAGCCT GTGGCACTGG gtgagcccag gccggggccg
```

Supplementary Fig.2. Human *GNMT* gene sequence from promoter region – 1320 bp to + 240 bp (intron 1) is shown. The sequence was retrieved from the UCSC hg19 human genome assembly (http://genome.ucsc.edu). Boxes indicate the three predicted AREs. Exon 1 is shown in uppercase letters, with sequences 5’ to the transcription start and intron 1 sequences shown in lowercase letters. The 5’ untranslated region (UTR) is indicated in blue and the coding region in red. +1 indicates the transcription start site.
