# Supplementary material for: Characterisation of the androgen regulation of glycine N-methyltransferase in prostate cancer cells
Source: J Mol Endocrinol. 2013 Aug 30;51(3):301–12. doi: 10.1530/JME-13-0169 (PMC3821059; doi:10.1530/JME-13-0169)
Supplement: Supplemental Data [file supp_JME-13-0169_Supplementary_table_1.pdf]

Supplementary Table 1

Mutagenesis primers used for generating the GNMTp-ARE mutants

Primer	Sequence (5' – 3')
GNMTp (ARE-I*)	CGCCAAAATATGTTAACGGTAGAGGA**ACGCGT**GGTTCTTGGCATCTTGAAC
GNMTp (ARE-II*)	GTTTTTTGCTTTTTGTTTTTTGAGACGGAGTCT**ACGCGT**GTTGCCCAGGCTGGAG
GNMTp (ARE-III*)	GCAGGATGGTGGACAGCG**ACGCGT**GGACCCGCTCCCTGGGGG

The restriction sites are in bold and underlined.
