# Supplementary material for: Characterisation of the androgen regulation of glycine N-methyltransferase in prostate cancer cells
Source: J Mol Endocrinol. 2013 Aug 30;51(3):301–12. doi: 10.1530/JME-13-0169 (PMC3821059; doi:10.1530/JME-13-0169)
Supplement: Supplemental Data [file supp_JME-13-0169_Supplementary_table_2.pdf]

Supplementary Table 2

TaqMan Assay-on Demand primers (Applied Biosystems)

Gene	Assay Identification N.
GNMT	Hs00219089_m1
AR	Hs00171172_m1
PSA	Hs00377590_s1
NDRG1	Hs00608389_m1
TMPRSS2	Hs00237175_m1
