# Supplementary material for: Characterisation of the androgen regulation of glycine N-methyltransferase in prostate cancer cells
Source: J Mol Endocrinol. 2013 Aug 30;51(3):301–12. doi: 10.1530/JME-13-0169 (PMC3821059; doi:10.1530/JME-13-0169)
Supplement: Supplemental Data [file supp_JME-13-0169_Supplementary_table_3.pdf]

Supplementary Table 3

Oligonucleotide sequences used for the EMSA assay

ARE	Name	Sequence (5' – 3')
GNMT-ARE wild-type	ARE_{GNMT}	ATGGTGGACAGCGTGTACCGGAC
GNMT-ARE mutant	ARE_{GNMT*}	ATGGTGGACAGCGACGCGTGGAC
PSA-ARE I	ARE_{PSA}	TTGCAGAACAGCAAGTGCTAGCT
