# Supplementary material for: Characterisation of the androgen regulation of glycine N-methyltransferase in prostate cancer cells
Source: J Mol Endocrinol. 2013 Aug 30;51(3):301–12. doi: 10.1530/JME-13-0169 (PMC3821059; doi:10.1530/JME-13-0169)
Supplement: Supplemental Data [file supp_JME-13-0169_Supplementary_table_4.pdf]

Supplementary Table 4

ChIP primers for real-time PCR

Primer	Sequence (5' – 3')
Forward (PSA enhancer)	TGACAGTAAACAAATCTGTTGTAAGAGACA
Reverse (PSA enhancer)	AGCAGGCATCCTTGCAAGAT
Forward (PSA negative)	TCCACTCCAGCTCTAAGATGGT
Reverse (PSA negative)	CAGGTAAACTCCAAGCACAGTGA
Forward (GNMT ARE)	AGGATGGTGGACAGCGTGTA
Reverse (GNMT ARE)	AAGCAGCCATGCCTTGTACT
